# Epidemiological Assessment of Eight Rounds of Mass Drug Administration for Lymphatic Filariasis in India: Implications for Monitoring and Evaluation

**DOI:** 10.1371/journal.pntd.0001926

**Published:** 2012-11-29

**Authors:** Subramanian Swaminathan, Vanamail Perumal, Srividya Adinarayanan, Krishnamoorthy Kaliannagounder, Ravi Rengachari, Jambulingam Purushothaman

**Affiliations:** Vector Control Research Centre, Indian Council of Medical Research, Indira Nagar, Pondicherry, India; University of Kelaniya, Sri Lanka

## Abstract

**Background:**

Monitoring and evaluation guidelines of the programme to eliminate lymphatic filariasis require impact assessments in at least one sentinel and one spot-check site in each implementation unit (IU). Transmission assessment surveys (TAS) that assess antigenaemia (Ag) in children in IUs that have completed at least five rounds of mass drug administration (MDA) each with >65% coverage and with microfilaria (Mf) levels <1% in the monitored sites form the basis for stopping the MDA. Despite its rigour, this multi-step process is likely to miss sites with transmission potential (‘hotspots’) and its statistical assumptions for sampling and threshold levels for decision-making have not been validated. We addressed these issues in a large-scale epidemiological study in two primary health centres in Thanjavur district, India, endemic for bancroftian filariasis that had undergone eight rounds of MDA.

**Methodology/Principal Findings:**

The prevalence and intensity of Mf (per 60 µl blood) were 0.2% and 0.004 respectively in the survey that covered >70% of 50,363 population. The corresponding values for Ag were 2.3% and 17.3 Ag-units respectively. Ag-prevalence ranged from 0.7 to 0.9%, in children (2–10 years) and 2.7 to 3.0% in adults. Although the Mf-levels in the survey and the sentinel/spot check sites were <1% and Ag-level was <2% in children, we identified 7 “residual” (Mf-prevalence ≥1%, irrespective of Ag-status in children) and 17 “transmission” (at least one Ag-positive child born during the MDA period) hotspots. Antigenaemic persons were clustered both at household and site levels. We identified an Ag-prevalence of ∼1% in children (equivalent to 0.4% community Mf-prevalence) as a possible threshold value for stopping MDA.

**Conclusions/Significance:**

Existence of ‘hotspots’ and spatial clustering of infections in the study area indicate the need for developing good surveillance strategies for detecting ‘hotspots’, adopting evidence-based sampling strategies and evaluation unit size for TAS.

## Author Summary

Lymphatic filariasis is a mosquito-borne parasitic disease caused by filarial worms. Over 120 million people are infected and 43 million have chronic disease. Since 2000, most of the endemic countries launched mass drug administration (MDA) programme with a goal to eliminate the disease as a public health problem by 2020. MDA impact is monitored by assessing microfilaraemia (Mf) prevalence from a few sites and when it drops to <1% after 5 rounds of MDA each with >65% coverage, transmission assessment surveys (TAS) are carried out to decide on stopping further MDA. We used a mass blood survey in two primary health centres to assess the epidemiological situation after 8 rounds of MDA and evaluated the soundness of conclusions drawn from monitoring a few sites for initiating TAS. Mf-prevalence was 0.2%, both in the survey and monitoring sites, justifying the need for TAS, although data on MDA coverage was not available. The antigenaemia-prevalence in children born during the MDA period was 0.8%, well below the level (<1%), required for stopping MDA. However, we identified a few sites with ongoing transmission (‘hotspots’) and spatial clustering of infection which indicate the need for good sampling/surveillance strategies to detect such ‘hotspots’.

## Introduction

The goal of the global programmes to eliminate lymphatic filariasis (GPELF) launched in 2000 is to eliminate the disease as a public health problem by 2020. The programme aims to achieve the goal by interrupting transmission through annual single dose mass drug administration (MDA) of albendazole (fixed dose 400 mg) either with diethylcarbamazine citrate (DEC, 6 mg/kg body weight) or ivermectin (150–200 µg/kg) for at least 5–6 years. By 2010 the programme had made significant progress in 53 of the 72 endemic countries, delivering billions of treatments to nearly 897 million people in the world [1]. India, which contributes to nearly one-third of the global burden of LF, aims to achieve LF elimination by 2015. The programme was initiated on a pilot basis in 13 of the 250 endemic districts (implementation unit, IU) during 1996–97 with DEC alone and thereafter with DEC and albendazole. The programme has been gradually scaled up to reach all the 250 endemic districts spread over 20 states and union territories covering nearly 421 of the 590 million people at risk in 2007 (www.nvbdcp.gov.in).

The impact of the programme is monitored once in two years in each IU by screening the population in at least one sentinel and one spot-checks sites for microfilaraemia (Mf) [2]. Several endemic countries reported that delivering 5–6 rounds of MDA reduced the prevalence of Mf and antigenaemia (Ag) in many IUs [3]. In India, it has been reported that in 192 of the 250 IUs Mf-prevalence has reached <1% – the recommended threshold for proceeding with further epidemiological assessment [3]. Such an assessment is required prior to making a decision to stop or continue MDA in these IUs.

The WHO monitoring and evaluation guidelines (2005) required a multi-step process to be completed before MDA could be stopped [2].The recommended sampling strategy for stopping MDA in an IU is to test (i) by a cluster design based lot quality assurance-cluster sampling (LQAS) of 10 young children (age-class depends on number of rounds of MDA) from each of 30-clusters (communities) for antigenaemia (Ag) status and (ii) if no positives are found, an additional LQAS of 3000 school entrants drawn from the entire IU for Ag-status. MDA has to be stopped if no Ag-positives are detected by (ii). The above procedure is applicable only when (a) the Mf-prevalence is <1% in both sentinel and spot check sites (b) Mf-prevalence is <1% in 5–10 additional sites of high risk transmission, and (c) Ag is negative among children aged 2–4 years in all these sites. Although this rigorous process was designed to ensure that transmission was stopped with a high degree of probability, it was cumbersome. Therefore, the WHO has revised the guidelines in 2011 [4] and proposed transmission assessment surveys (TAS) for making decision to stop/continue MDA. As per the revised guidelines the recommended Ag-threshold for TAS is a defined number of Ag-positive children (depends on the target population size) such that there is at least a 75% chance of stopping MDA if the true prevalence is less than 1.0% and no more than a 5% chance of stopping MDA (incorrectly) if the true prevalence is ≥2%. TAS are to be carried out in IUs that have completed at least five rounds of MDA, each with >65% coverage and with Mf-prevalence of <1% in both sentinel and spot check sites. However, both the old and the revised guidelines, suggested cluster design for the LQAS, which is not based on empirical evidence about the distributions of infection at household and site level.

We carried out a study to address the above mentioned issues in two primary health centres (PHC), in Thanjavur district, India, known to be endemic for filariasis caused by periodic *Wuchereria bancrofti* and transmitted by *Culex quinquefasciatus* (National Vector Borne Disease Control programme, NVBDCP). A large proportion of the population of these two PHC areas, where eight rounds of MDA were completed, was screened for Mf and Ag. This extensive data set permitted us to draw critical epidemiological inferences. In the present communication we have examined age-specific infection patterns, distribution of infections by households and sites, and have identified ‘hotspots’ (please see ‘Methods’ below for definition). We also determined the transmission threshold level in terms of Ag-prevalence in children, as hitherto published results on threshold for stopping MDA are based on prevalence of Mf [5]–[8] and Ag [9] at community level. The findings of the study are discussed in the context of the WHO guidelines of 2005 [2] and 2011 for TAS to stop MDA [4] highlighting the importance of ‘hotspots’, size of the evaluation unit and threshold levels of antigen prevalence in children in such evaluations.

## Methods

### Study area

**Figure 1:**
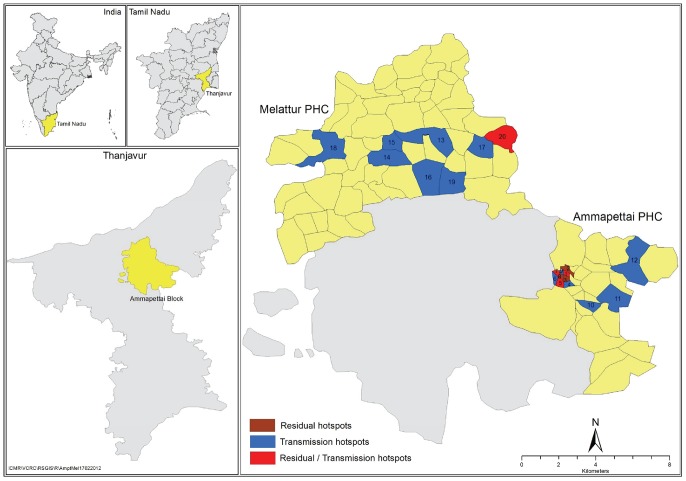
Map showing the study area along with ‘hotspots’.

This study was carried out in two PHCs, namely Ammapettai and Melattur (here after referred to as ‘evaluation unit’, EU) in Ammapettai block, Thanjavur district in Tamil Nadu State, India (Figure 1). There are 18 villages and 15 urban wards (hereafter referred as ‘sites’) under six health sub-centres (HSC) in Ammapettai PHC, and 62 villages under eight HSCs in Melattur PHC.

Thanjavur district (population: 2.2 million) was one of the 13 districts endemic for bancroftian filariasis, in the State of Tamil Nadu. MDA was initiated in 1997 in this district by the State government and subsequently brought under the national programme to eliminate LF in India. There are 58 PHCs and 309 HSCs in this district (IU). Eight rounds of MDA had been completed in this district by the time the study was initiated in 2008. While DEC alone was given in the years 1997, 1999, 2000, and 2004, it was co-administered with albendazole in the years 2001, 2002, 2003, and 2007. MDA was not carried out in 1998, 2005 and 2006. Drugs were distributed by Filaria Assistants drawn from the community under the supervision of local health personnel in each PHC, by visiting 250 persons each. Pregnant women and children <2 years were excluded from MDA. Coverage and compliance as assessed by an independent agency (Vector Control Research Centre) ranged from 55.8 to 91.2%, and 34.3 to 53.4% respectively during 2001 to 2010. Compliance in this document refers to surveyed coverage as indicated in the revised WHO guidelines for TAS [4]. Epidemiological data in 2000 indicates that the overall prevalence of microfilaria (Mf) was 2.6%, with 11 out of 14 health blocks showing >1% prevalence (Vector Control Research Centre, unpublished observations). The Mf surveys carried out under the programme, prior to each round of MDA in sentinel and spot check sites, showed a declining trend in Mf-prevalence (0.16%, 0.15%, 0.15%, 0.0% and 0.09% during 2005, 2006, 2007, 2008, and 2009 respectively), and it was below 1% in all the 16 sentinel and spot check sites after the 2004 MDA (Vector Control Research Centre, unpublished observations).

### Demographic registration

In the present study, a hand held personal digital assistant (PDA) was used in the field to collect demographic details that included name, age and gender of family members. A barcode label was used to identify each person. The barcode label was pasted against the name of each person in a register and the barcode was scanned by a Bluetooth scanner attached to the PDA. The PDAs equipped with GPS devices (global positioning system) were used to capture geographical coordinates (latitude, longitude, altitude) of the houses located in the study area. The data from each PDA were synchronized in the field to create a master database. At the end of each day, data were uploaded to a field laptop computer in which the data was managed using SQL server (2005, Microsoft Corporation) and a backup of the data was created on an external hard disk. Digital maps were made for all the sites (villages/wards) using their geographical coordinates.

### Mass screening for Mf and Ag

Mass screening was done by house visits between 1930 hrs and 2300 hrs to assess Mf and Ag-status. The survey was carried out between October 2008 and July 2009. While screening, identity of a person (Person ID) in the PDA was verified with the family members. Glass slide and vial for that person were labeled using barcodes and were scanned against the person ID registered in the PDA. After obtaining written informed consent (from parents for the children below 15 years of age) finger-prick blood samples were collected for detection of Mf and Ag. About 60 µl blood was drawn onto a glass slide to make thick smear for Mf and 200 µl blood into a 2 ml round bottom vial for Ag-assay. All the available and consenting persons in each house during the visit were screened. Repeated visits to all the sites were made to increase the participation of the population in the microfilaraemia and antigen screening.

Blood smears were dehaemoglobinized, dried, fixed in acidified methyl alcohol and stained with JSB-1 stain. Serum from the blood sample was separated using refrigerated centrifuge for Ag-assay. The serum samples were preserved at −40°C in the Field Laboratory at Thanjavur. At weekly intervals both the smears and serum vials were transported to the Headquarters of the Vector Control Research Centre located at Pondicherry (∼200 kms away from Thanjavur) for further processing/examination. Blood slides were examined under microscope and the numbers of Mf found were recorded. 10% of slides selected randomly were cross-examined for quality control. The serum samples (of 100 µl) were used to measure quantitative filarial antigen levels by the Og4C3 enzyme linked immunosorbent assay (ELISA, TropBio, Townsville, Australia). The ELISA was done with boiled plasma diluted 1∶4, a negative control, and 7 standard samples with increasing concentrations of *O. gibsoni* antigen, as recommended by the manufacturer. Optical density readings of the seven manufacturer standards were plotted for each microtitre plate and were converted to antigen units, with values ranging from 0 (standard sample 1) to 32000 (standard sample 7). The Ag-units for the study subjects were derived from the standard curve. Serum samples with Ag-units ≥ standard 3 of the manufacturer (≥128 Ag-units) were considered positive for circulating filarial antigen. Results of slide examination and antigen assays were entered into a Microsoft Excel spreadsheet in which the demographic and mass screening database was imported from Microsoft SQL server.

### Ethical consideration

The research proposal was approved by the Institutional Human Ethics Committee of VCRC. Informed written consent was obtained from all adult participants and parents for children aged below 15 years to collect finger prick blood for Mf and Ag-assay. Consent also included collecting information on the clinical manifestations of filariasis, if any, by observing for lymphoedema of limbs and self-reporting of hydrocele. Confidentiality of the identity and results was maintained. Those found positive for Mf and/or Ag were treated with DEC (6 mg/kg body weight) daily for 12 days following the national programme guidelines of India. Special demonstrations were held for those with lymphoedema on limb hygiene and care. Those with hydrocele were advised to consult their doctors for further management.

### Hotspots

Sites where Mf-prevalence ≥1% (irrespective of Ag-status in children) were defined as ‘residual hotspots’ and the sites with at least one Ag-positive child in the age range 2–8 years were considered as ‘tranmission hotspots’.

### Statistical analyses

The prevalence (%) of Mf or Ag was calculated as the number of people with Mf or Ag divided by the number of people examined. The geometric mean Mf-intensity was estimated as the antilog of the (sum of [log(X+1)/N]−1). The geometric mean Ag-intensity was estimated as the antilog of the sum of [log(X)/N], where X is the Ag-units in each person, and N is the number of persons screened. Mean values of Mf and Ag across different age groups and genders were compared using analysis of variance (ANOVA) model on log-transformed values of (Mf+1) or Ag. Pair wise comparison was made using least significant difference (LSD). The heterogeneity chi-square test was used to compare the prevalence across gender and the chi-square test for trend in proportions to compare the prevalence across age. The 95% confidence interval (CI) for community prevalence of Mf and Ag were based on normal approximation to binomial distribution and that for antigen prevalence in children the exact binomial confidence intervals were used. Student's t- test for independent samples was used to compare the geometric mean intensity of Mf or Ag. Logistic regression analysis was done to examine the relationship between community Mf-prevalence (independent variable) and Ag-status (positive or negative, dependent variable) in children aged 2–8 years (born during the period of MDA). Receiver operating characteristic (ROC) curve analysis was used to determine the Ag-prevalence threshold in children at maximum values of sensitivity (probability of predicting an Ag-positive child as Ag-positive for a given cut-off -threshold- of community Mf-prevalence) and specificity (probability of predicting an Ag-negative child as Ag-negative for a given cut-off of community Mf-prevalence). Sensitivity and specificity were determined by comparing the values predicted by logistic regression with the observed ones. Poisson and negative binomial probability models were fitted to the observations at household or sites level to see if filarial infections are clustered in certain households or sites. Chi-square test was used to test the goodness of fit of the models. For all statistical significant tests, the probability of a Type I error was set at 5% (α = 0.05). All statistical analyses were carried out using the statistical software IBM SPSS v.19.0 and STATA version 9.0. Getis-Ord G-statistic was used to test the spatial clustering of ‘hotspots’ using ARC GIS version 9.3.

## Results

### Demographic profiles of the study population

A total of 50363 persons were enumerated from 12759 households (household size, range: 1–11; median: 3) distributed over 95 sites in two PHCs. The populations in different sites ranged from 128 to 2384. The age-profiles of the population are comparable between males and females with a male to female ratio of 1∶0.98. Children below 10 years constituted about 15% each of the male and female populations, with ∼5% of them being school entrants (aged 5–6 years). The population in the age-group 20–59 years constitute the work force in the study area, which is ∼56% of the male and female populations.

### Mass screening

People living in 11466 (90%) of the 12759 houses listed were contacted for screening. The remaining 1293 houses were found locked despite repeated visits. A total of 35582 persons participated in the mass-screening programme forming 70.7% of the population (50363). Among these, blood samples from 139 persons could not be collected for Mf. Samples from 1149 persons either could not be collected or were insufficient for Ag-assay. As a result, the number of persons from whom samples were collected for Mf, Ag and both was 35443 (70.4%), 34433 (68.4%) and 34294 (68.1%) respectively. Both males and females were equally represented (>70%) in all age classes except males in 21–30 and 31–40 years (Table 1), who were fewer than females.

**Table 1:** Age and gender specific participation rates in mass screening programme.

Age-group (Years)	Enumerated population			Screened population			Participation rate (%)		
	Female	Male	Total	Female	Male	Total	Female	Male	Total
<2	579	537	1116	21	15	36	3.6	2.8	3.2
2–5	1592	1456	3048	840	866	1706	52.8	59.5	56.0
6–7	835	839	1674	645	675	1320	77.2	80.5	78.9
8–10	1388	1309	2697	1134	1171	2305	81.7	89.5	85.5
11–20	5400	5046	10446	4072	4001	8073	75.4	79.3	77.3
21–30	5083	4367	9450	3575	2389	5964	70.3	54.7	63.1
31–40	4279	3503	7782	3548	2335	5883	82.9	66.7	75.6
41–50	3405	3320	6725	2707	2527	5234	79·5	76.1	77.8
>50	3813	3612	7425	2390	2671	5061	62.7	73.9	68.2
Total	26374	23989	50363	18932	16650	35582	71.8	69.4	70.7

### Epidemiological assessment

#### Overall prevalence and intensity

Mf and Ag-positive individuals were detected in 32 and 91% of the 95 sites respectively. The prevalence of Mf ranged from 0.0 to 3.9% (mean: 0.2%) and Ag from 0.0 to 8.2% (mean: 2.3%) in different sites. The geometric mean (95% CI) of Mf-intensity (per 60 µl blood) and Ag-intensity (units per 100 µl serum) were 0.004 (0.003–0.005) and 17.3 (17.1–17.5) respectively. The Ag-intensities among Mf-positive (n = 75) and Mf-negative individuals (n = 34219) were 24350.3 and 16.9 units respectively. The difference in Ag-intensities between Mf-positives and Mf-negatives was significant (t = 65.5, P<0.001).

#### Age-and gender-specific prevalence

**Figure 2:**
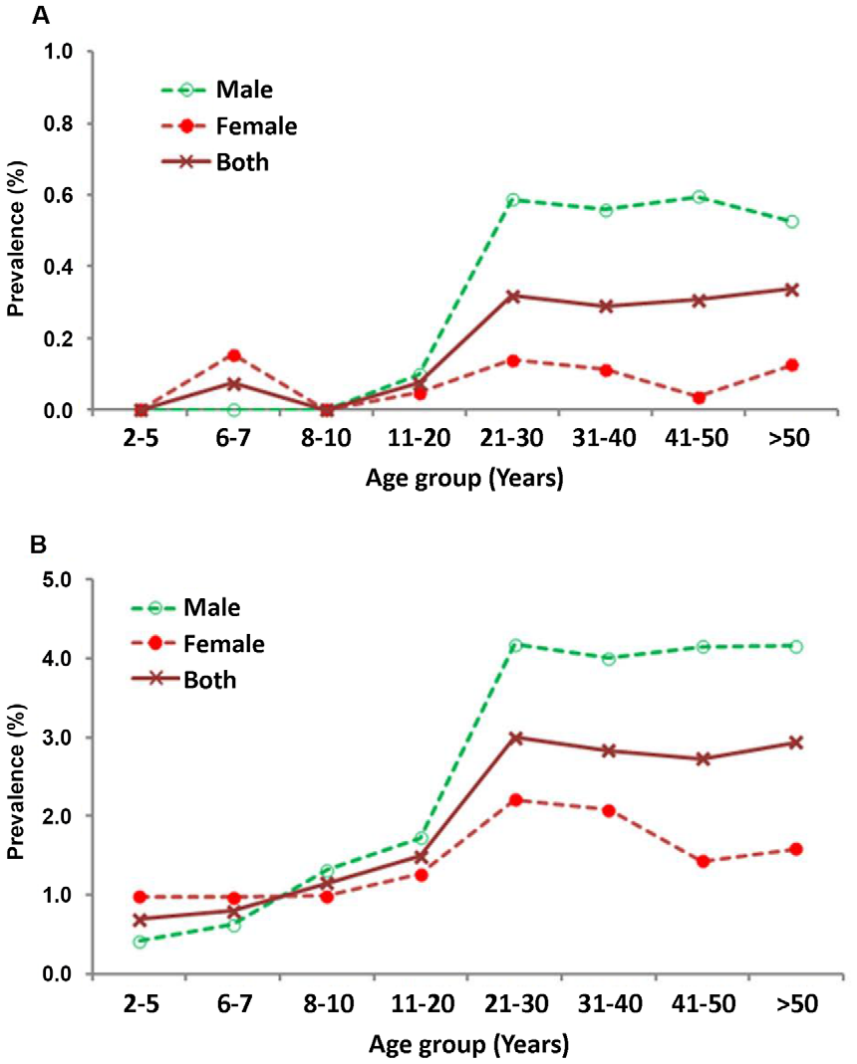
Age and gender-specific prevalence of microfilaraemia (A) and antigenaemia (B).

Figure 2 illustrates the pattern of age-specific prevalence of Mf and Ag. The overall prevalence (genders combined) of Mf is below 0.1% up to 20 years and thereafter it fluctuated between 0.3 and 0.4%. The prevalence of Ag was nearly constant (range: 0.69% to 0.85%) in the age range 2–10 years, reached a peak of ∼3% at 21–30 years and thereafter it remained almost constant (range: 2.7–3.0%). Neither the Mf-prevalence nor the Ag-prevalence differed significantly within two broad age-ranges (2–20 years and >20 years for Mf-prevalence; 2–10 years and >20 years for Ag-prevalence, P>0.1 for all comparisons within each age-range). However, the increase in Ag-prevalence between 2–10 and 11–20 years (P<0.006) as well as between 11–20 and 21–30 years (P<0.001) of age was significant. A similar comparison between two broad age-groups (≤20 years and >20 years) also showed that both the prevalence of Mf (0.05% vs 0.31%) and Ag (1.28 vs 2.88%) were significantly higher among persons aged >20 years than that in those aged ≤20 years (P<0.001 for both comparisons).

Figure 2 also shows gender specific prevalence of Mf and Ag. In the age range of 2–10 years only one female child (6 years) was microfilaraemic. However, in the same age-range, Ag was detected in both males (prevalence, range: 0.4 to 0.7%) and females (∼1%). Statistical comparisons of the prevalence revealed that the gender differences was not significant in the above age sub-groups (P>0.2 for all comparisons) suggesting that acquiring new infection is independent of age and gender. The prevalence of both Mf and Ag started increasing in both genders at 11–20 years to reach a peak at 21–30 years. In the ages above 30 years, while both the prevalence profiles were similar for males, they declined for females exhibiting a convex relationship with age. Further, both Mf and Ag-prevalence profiles for adult males were at higher levels than the respective profiles of females. Statistical analysis showed that adult males (>20 years) had significantly higher prevalence of Mf and Ag as compared to their female counterparts for all age sub-groups (P<0.05).

#### Age and gender specific intensity of infection (Mf/Ag)

**Figure 3:**
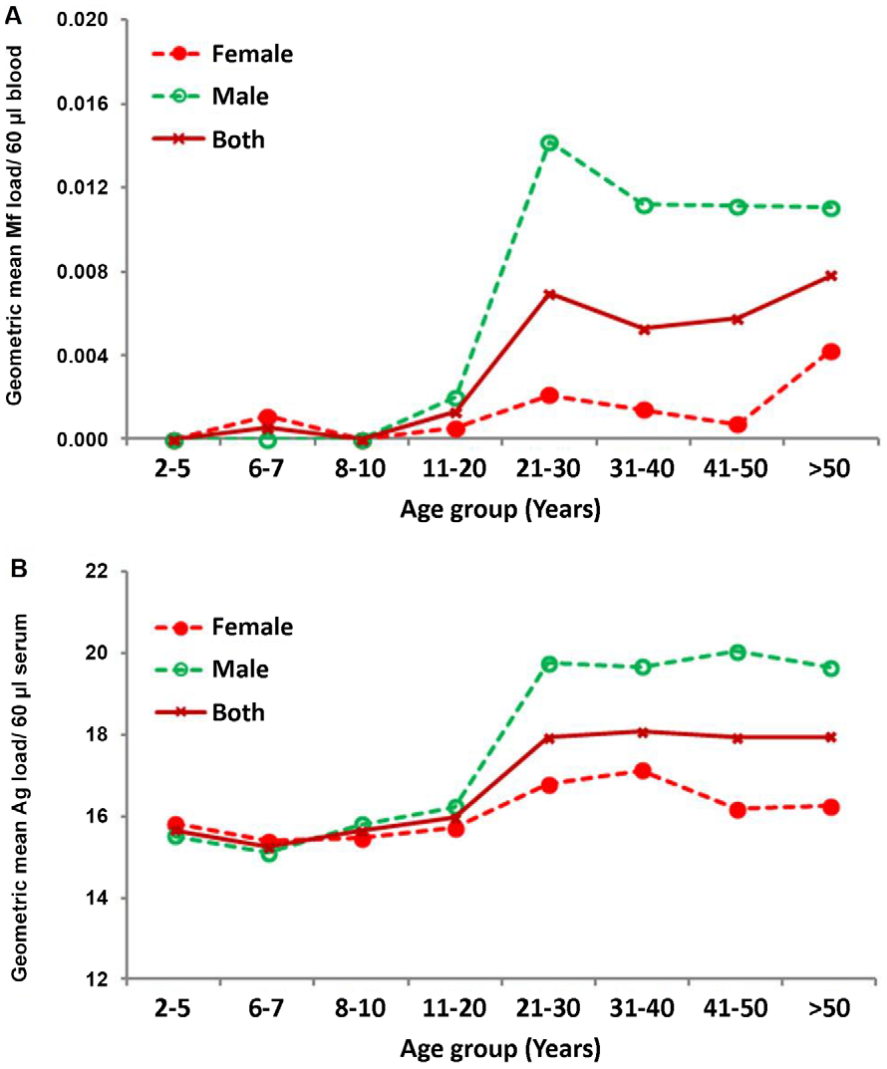
Age and gender-specific geometric mean microfilaria (A) and antigen (B) intensity.

The overall (gender combined) Mf-intensity was almost zero per 60 µl up to 20 years. Thereafter the intensity fluctuated between 0.007 and 0.008 (Figure 3), but was not statistically significant (P>0.3 for all pairwise comparisons). However, a significant (t = 4.89, P<0.001) increase was observed in adult age-groups (0.006 per 60 µl) when compared to that of persons aged ≤20 years (0.001 per 60 µl). The Ag-intensity was almost constant up to 20 years of age (16 units per 100 µl serum) and thereafter it reached and stabilized at the level of 18 units. The difference in Ag-intensity among those aged ≤20 years and >20 years was significant (t = 11.4, P<0.001).

The gender specific age-intensity profiles were almost zero per 60 µl blood for Mf and 16 units per 100 µl serum for Ag up to 20 years of age (Figure 3). Thereafter, the Mf-intensities leveled off at 0.014 and 0.002 for males and females respectively and the corresponding levels of Ag-intensities were 20 and 17 units. While both Mf and Ag-intensities were independent of gender in persons aged <20 years, significant differences were observed between genders in all adult age-subgroups (21–50 years, P<0.05 for all pairwise comparisons). Among all Ag-positive individuals (n = 784), the Ag-intensity increased from 2742 at 2–5 years to reach a peak 6535 units at 21–30 years and thereafter it stabilized around 4500–5800 Ag-units.

#### Distribution of filarial infection by households and sites

**Figure 4:**
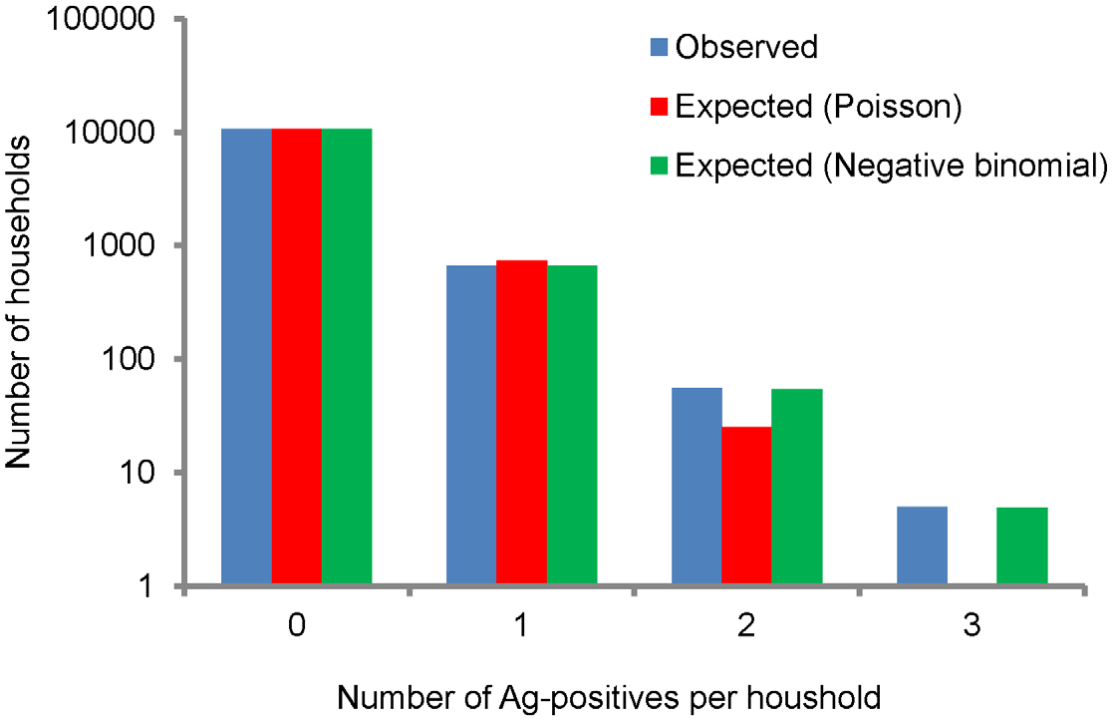
Observed and expected number of households with antigen positives by Poisson and negative binomial models.

The distribution of persons in terms of antigen status by households is given in Figure 4. A total of 11375 households were covered in the mass screening survey for Ag-detection. Of them in a majority (93.7%) of the households there were no persons with filarial infection. There was one Ag-positive person in 5.8% of the households. Only 0.5% had ≥2 Ag-positives. Fitting of Poisson (random) and negative binomial (over-dispersed) models to the data suggested that the numbers of households expected by negative binomial model (χ^2^ = 0.05, P = 0.8) is in better agreement with observations than by the Poisson model (χ^2^ = 55.8, P<0.001, Figure 4), suggesting that the Ag-cases are aggregated (negative binomial aggregation parameter, k = 0.6) in households.

**Figure 5:**
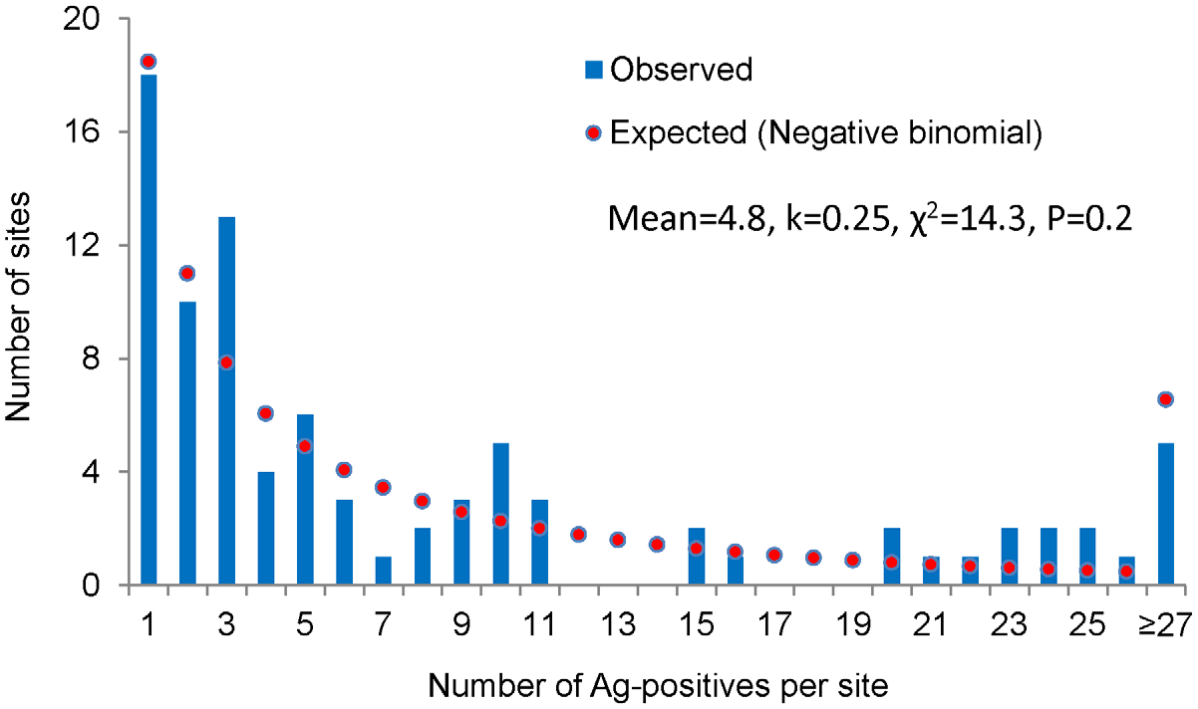
Observed and expected number of sites with antigen positives by zero-truncated negative binomial model.

The frequency distribution of Ag-positives by sites is shown in Figure 5. About 68% of the sites had only 1–10 Ag-positives and 23% of the sites had more than 10 positives, showing a skewed distribution. This was further confirmed by good fit of the zero-truncated negative binomial model (χ^2^ = 14.3, P = 0.2). The small value of the aggregation parameter ‘*k*’ (0.25) of the fitted model indicates that the Ag-positives are highly aggregated in a few of the sites.

### Hotspots

**Figure 6:**
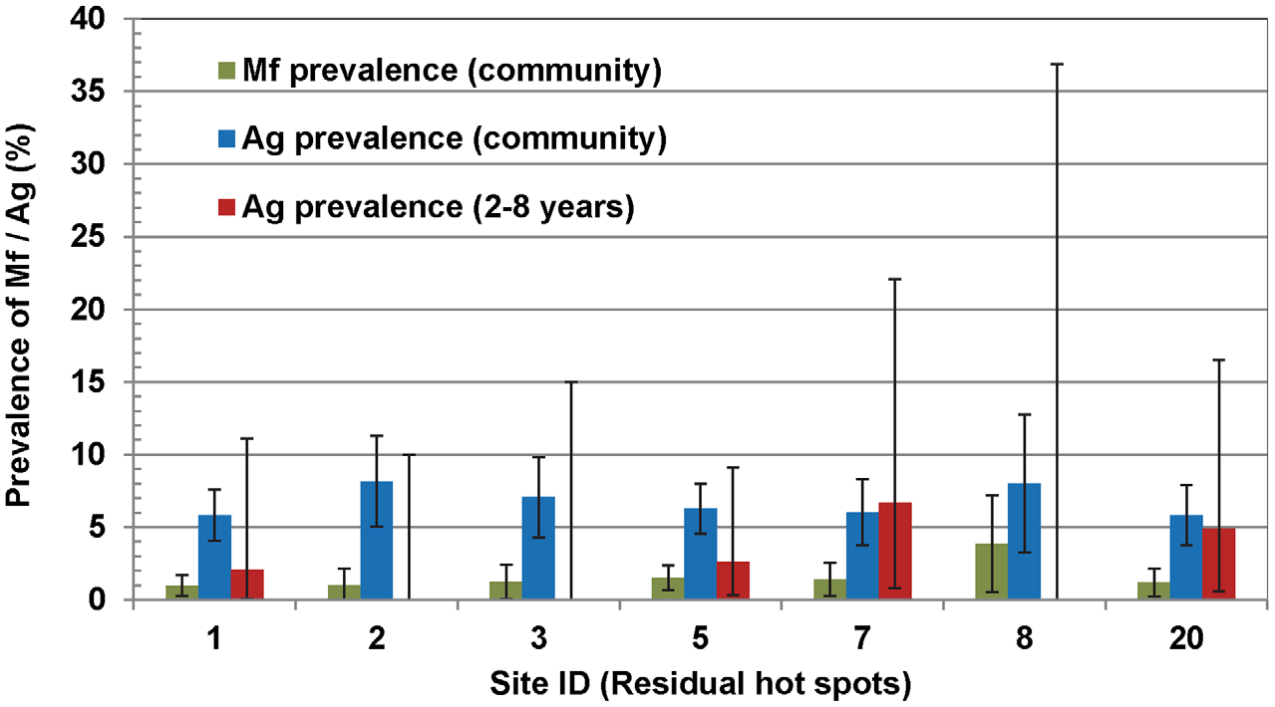
Prevalence of microfilaraemia (Mf) and antigenaemia (Ag) in the residual hotspots. Error bars are 95% confidence intervals.

The prevalence of Mf was ≥1% in seven (‘residual hotspots’) of the 95 sites. In these seven sites the Mf-prevalence ranged from 1.0 to 3.9% (mean: 1.3%; 95% CI: 0.9–1.7%) and Ag-prevalence from 5.0 to 8.2% (mean: 6.4%; 95% CI: 5.5–7.2%) (Figure 6). In the remaining 88 sites, the Mf-prevalence ranged from 0 to 0.8% (mean: 0.1%; 95% CI: 0.07–0.15%) and the Ag-prevalence from 0 to 7.1% (mean: 1.9%; 95% CI: 1.7–2.0%). In 8, 51, 26, and 3 of these sites Ag-prevalence was 0%, 0.2–2%, 2–5% and >5% respectively. In 4 of 7 residual hotspots, at least one Ag-positive child aged 2–8 years (born during the period of MDA) was detected (hence termed as ‘residual and transmission hotspots’) and the prevalence of Ag in this age-group from these four sites ranged from 2.1 to 6.7% (mean: 2.7%; 95% CI: 0.7–4.6%). Of the 88 sites (other than residual hotspots), 13 were detected as ‘transmission hotspots’. Ag-prevalence among children (2–8 years) in these sites ranged from 1.7–6.3% (mean: 2.6%; 95% CI: 1.4–3.8%). Altogether there were 17 ‘transmission hotspots’ in which the prevalence of Mf and Ag at community level and Ag-prevalence in children varied from 0 to 1.5% (mean: 0.5%; 95% CI: 0.3–0.6%), 0.3 to 6.5% (mean: 3.9%; 95% CI: 3.5–4.3%) and 1.7 to 6.7% (mean: 2.9%; 95% CI: 1.8–4.1%) respectively. While the community prevalence of both Mf (χ^2^ = 58.7, P<0.0001) and Ag (χ^2^ = 85.8, P<0.0001) varied significantly among the ‘transmission hotspots’, the Ag-prevalence in children did not vary significantly (χ^2^ = 5.7, P = 0.99).

Of the 3544 children screened either for Mf and/or Ag in the study area, 3528 were examined for both Mf and Ag and one child was found Mf-positive but Ag-negative, and the rest were Mf-negative. The Ag-level of the Mf-positive child was 16.3 units and the Ag-intensity among Mf-negative children (irrespective of Ag-status) was15.4 units. Among the Mf-negatives, the Ag-intensity was significantly (t = 62.0, P<0.001) higher in Ag-positives (n = 25, intensity: 2279.5 units) than that of Ag-negatives (n = 3502, intensity: 14.9 units).

At community level in the ‘transmission hotspots’, the Ag-intensities for Mf-positive (19953 units, n = 39) and Mf-negative (19.5 units, n = 8368) persons differed significantly (t = 35.9, P<0.001). Further, the intensity in the ‘hotspots’ (20.4 units for all persons irrespective of Mf-status, n = 8407) was significantly higher than that was observed in the remaining 78 sites (16.2 units, t = 17.4, P<0.001).

Data on community characteristics of the of the ‘hotspots’ (Table 2) showed that while all the ‘residual’ and two-third of the ‘residual and transmission’ hotspots are located in the town of Ammapettai PHC, most of the ‘transmission hotspots’ are located in rural villages of both PHC. Figure 1, also shows the spatial distribution of ‘residual’ (red), ‘transmission’ (blue) and the combination of both ‘residual and transmission’ (green) hotspots in the study area. Spatial clustering was observed among the residual and transmission hotspots. While ‘residual’ or ‘both residual and transmission hotspots’ were clustered in Ammapettai PHC, ‘transmission hotspots’ were clustered in both the PHCs. Spatial analysis based on Getis-Ord G-statistic indicated one significant spatial cluster of ‘residual hotspots’ in Ammapettai PHC (sites with high values of Mf-prevalence clustered together; Getis-Ord G = 0.49, Z = 7.54, P<0.0001) and two clusters of ‘transmission hotspots’ one at each PHC (sites with high values of Ag-prevalence clustered together, Getis-Ord G  = 0.13, Z = 3.2, P = 0.0012).

**Table 2:** Community characteristics of hotspots in the study area.

Site ID	Site name	No. of persons	No. of houses	Family size	Type of Locality	Nature of hotspots
1	Ammapettai -1	927	224	4	Town	Residual & transmission
2	Ammapettai Ward I	470	113	4	Town	Residual
3	Ammapettai Ward III	502	144	3	Town	Residual
4	Ammapettai Ward V	778	213	4	Town	Transmission
5	Ammapettai Ward XI	1109	339	3	Town	Residual & transmission
6	Ammapettai Ward XII	856	236	4	Town	Transmission
7	Ammapettai Ward XIII	663	170	4	Town	Residual & transmission
8	Ammapettai Ward XIV	216	52	4	Town	Residual
9	Ammapettai Ward XV	464	93	5	Town	Transmission
10	Puthur	869	241	4	Rural	Transmission
11	Puthur Nadupatti	768	210	4	Rural	Transmission
12	Ukkadai	1164	326	4	Rural	Transmission
13	Kallarnatham	871	164	5	Rural	Transmission
14	Melakulakkarai	287	69	2	Rural	Transmission
15	Melattur	2318	609	4	Town	Transmission
16	Narasingamangalam	344	92	4	Rural	Transmission
17	Neduncherry	252	58	4	Rural	Transmission
18	Perumakkanallur	686	149	5	Rural	Transmission
19	Ranganathapuram	513	118	4	Rural	Transmission
20	Thirukarugavur	743	229	3	Rural	Residual & transmission

### Estimation of transmission threshold

**Figure 7:**
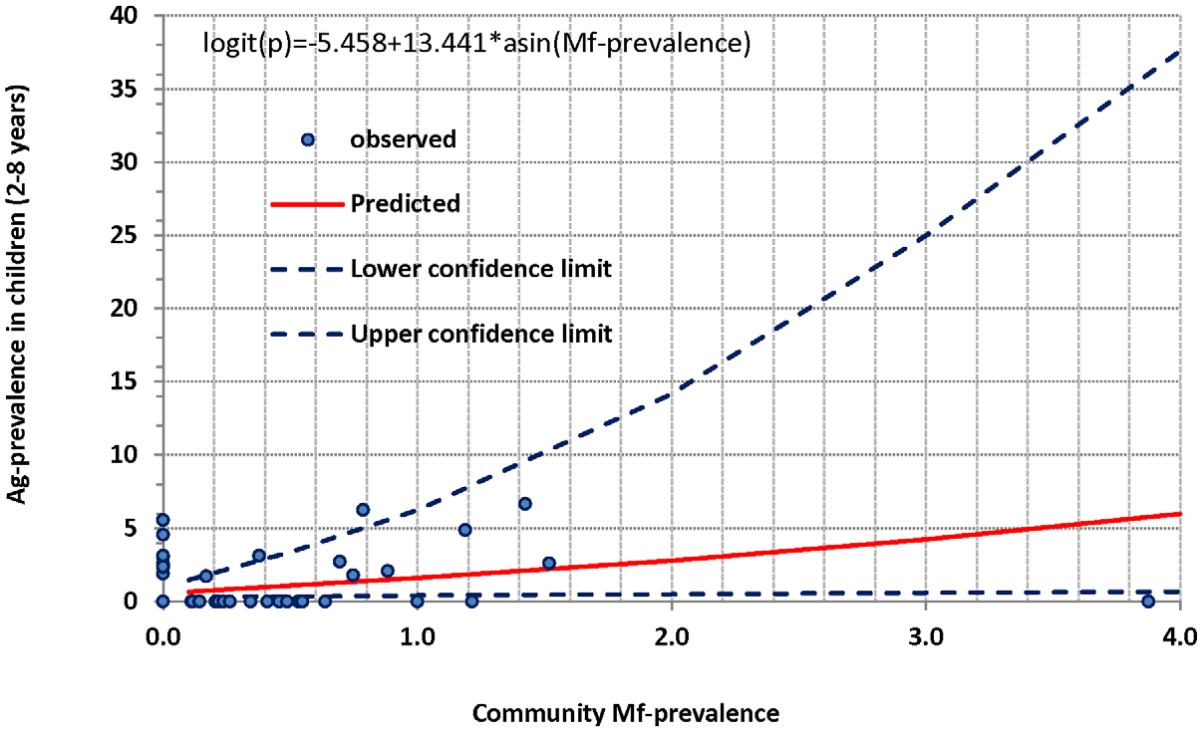
Relationship between community Mf-prevalence and Ag-prevalence in children (2–8 years) based on logistic regression analysis.

Ag-status in children aged 2–8 years and community Mf-prevalence from all the 95 sites were used to examine their relationship using logistic regression analysis and to estimate the transmission threshold in terms of Ag-prevalence in children. The logistic regression analysis revealed that the prevalence of Mf at community level is a significant predictor of Ag-status in children (Figure 7; χ^2^ = 8.54, P = 0.0035; Hosmer and Lemeshow goodness of fit, χ^2^ = 5.1, P = 0.16). The estimated relationship was used to predict the Ag-prevalence corresponding to Mf-prevalence of 1% (threshold) in the community and it was found to be 1.6% (95% CI: 0.4–6.2%). The sensitivity and specificity of the model (logistic regression) corresponding to the Ag-prevalence of 1.6% were 25% (95% CI: 10.6–47.1%) and 94% (95% CI: 93.2–94.8%) respectively. ROC analysis revealed that the sensitivity (45.8%; 95% CI: 26.2–66.8%) and specificity (81%; 95% CI: 79.9–82.5%) of the model could be maximum at an Ag-prevalence of ∼0.97%, which corresponds to 0.4% Mf-prevalence at community level.

## Discussion

### Epidemiological assessment

The epidemiological data generated in the present study through mass screening (∼70% of the population) provided an opportunity to validate one of the pre-TAS conditions and TAS. The overall Mf-prevalence (0.2%) in the study area is in agreement with that estimated from sentinel and spot-check sites for the IU (<1%). However, the study area has no historical data on MDA coverage and compliance excepting data available for two sites in the study area, which were part of an independent assessment survey carried out during the first round of MDA with co-administration of DEC plus albendazole in 2001 by the Vector Control Research Centre. This data provides an overall coverage and compliance of 66.4 (62.7 & 86.3% for the sites) and 65.0% (61.7 & 81.3% for the sites) respectively. If we consider the pre-TAS criterion, the study area will not qualify for implementing TAS, unless we assume similar compliance levels in subsequent MDA rounds. If an EU is a part of the IU as in the present case, the pre TAS criteria of IU can be considered applicable for the EU. Since the compliance was not consistently >65% in the IU, the study area will not qualify for TAS. Thus, lack of data on compliance or inadequate compliance will be a major constraint for implementing TAS and consequently MDA should continue until the criterion on compliance is met. For many such IUs, this will be a programmatic challenge for TAS in spite of meeting other two pre-TAS conditions. A similar situation may arise when IUs are combined to form a single EU.

**Figure 8:**
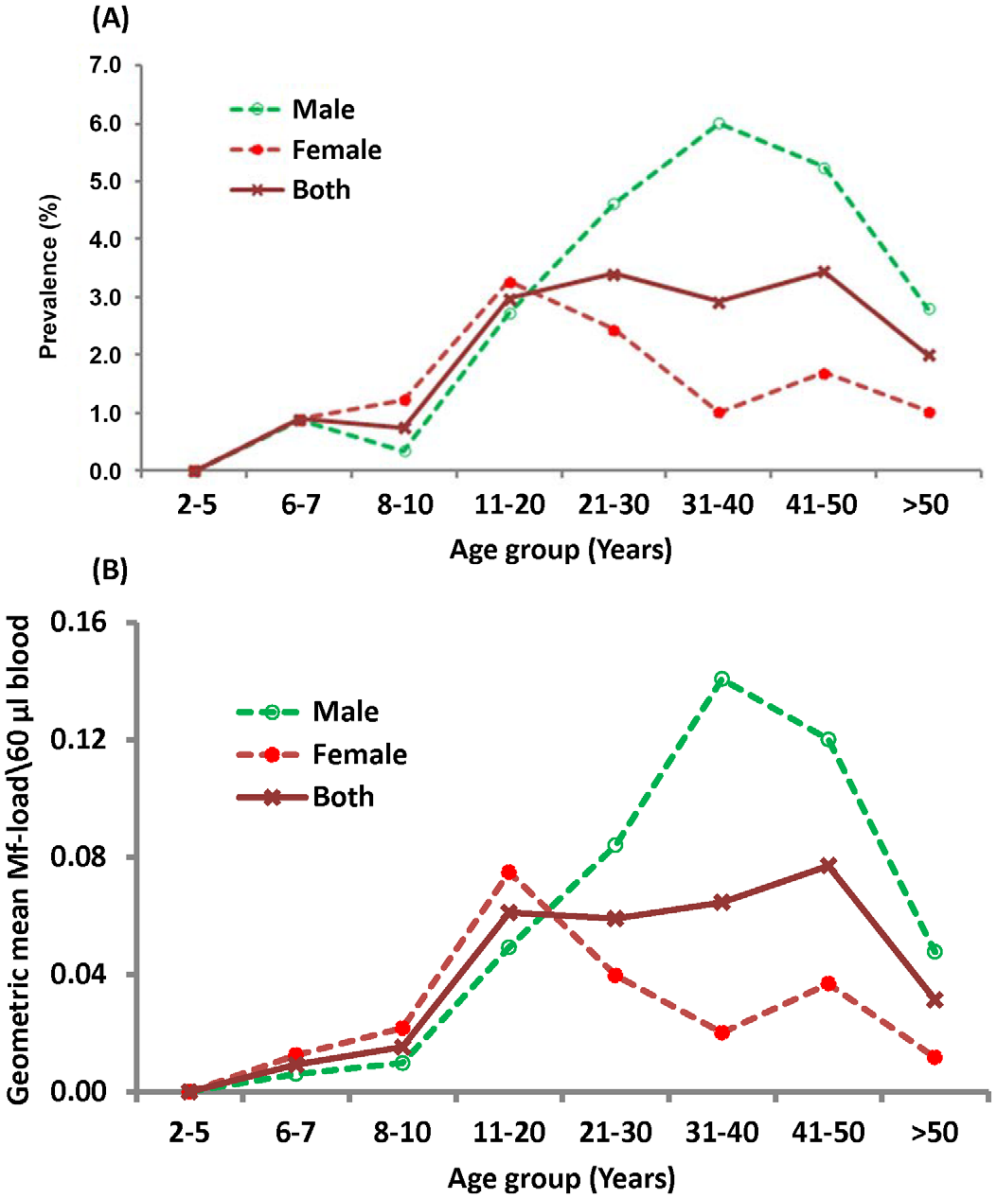
Age and gender-specific prevalence (A) and intensity (B) of microfilaraemia prior to MDA in Thanjvur district (IU). Source: Vector Control Research Centre (unpublished data).

A comparison of the observed patterns of the age-and gender specific prevalence of Mf and Ag, and intensity of Mf of the study area with that of the IU provides an idea on the extent of reductions that might have been achieved in the study area. The observed patterns of age and gender specific Mf and Ag-prevalence (Figure 2) are qualitatively similar but quantitatively at lower levels than those observed prior to MDA (Figure 8A; overall Mf-prevalence 2.6%) in the IU as well as elsewhere without any intervention [10]–[19]. Similar observations have been made from age and gender-specific Mf-intensity profiles (Figure 3A & Figure 8B). Reduction in Mf-intensity (almost ‘zero’ in persons aged<20 years and about ten-fold reduction in persons aged >20 years for both genders, Figure 3) following eight rounds of MDA in the study area when compared to the corresponding profiles during pre-MDA for the IU (Figure 8B) could be attributed to MDA. The Ag-prevalence (0.8%) in children (Figure 2) is well below 1% which is the level recommended in the target age class (6–7 years) for stopping MDA under the revised TAS guidelines [4].

The Ag-intensities among Mf-positive persons were significantly higher in the entire study area (24950 units) as well as in the ‘transmission hotspots’(19953 units) compared to that among Mf-negatives (16.9 for the entire study area and 19.5 units for the ‘transmission hotspots’) indicating that a large number of infections are required to become microfilaraemic. In the ‘transmission hotspots’ the Ag-intensity (20.4 units; irrespective of Mf-status, residual Ag-level) was significantly higher than that was observed in the remaining sites (16.2 units). However, the Ag-intensity of the 25 Ag-positive children (2–8 years) was much lower (2280 units) than that of the Ag-intensities that were observed among all the Mf-positives either in the ‘transmission hotspots’ or in the entire study area. These Ag positive children in the ‘transmission hotspots’ are either in pre-patent period (acquired infections in the recent past) who may become microfilaraemic over a period of time or having worms which are not sufficient enough to mate and produce Mf or some of them may be false-negative for Mf.

Assuming that the study area meets the pre-TAS criteria, the Ag-prevalence data of the present study was used for validating the revised TAS [4] for making decision on stopping/continuing MDA. The results of screening 1259 children (75.2% of the target population of 1674) in the age class 6–7 years, covering all the 95 sites in the study area, showed 10 Ag-positives. Despite large number of children examined than that required for the TAS (n = 891), the number of Ag-positives were lower than that the critical cut-off (d = 11) required for this age-class [4], suggesting that the study area passed the TAS.

### Hotspots

Another objective of the study was to examine the soundness (accuracy/validity) of the conclusions drawn from monitoring a few sentinel- and spot-check sites for initiating TAS [2], [4]. Though our findings are in conformity with the results (Mf-prevalence <1% after 8 rounds of MDA) of sentinel and spot check sites in the IU, we detected ≥1% Mf-prevalence in seven sites (‘residual hotspots’). Similar situations, although based on sample surveys, have been reported in India, where 49–84% of the population have undergone either six rounds of MDA with DEC plus albendazole or 9/10 rounds of MDA with DEC/ivermectin [7], [20]. In Nigeria, five of the 10 sentinel sites were found to remain as ‘residual hotspots’ despite 6–10 rounds of MDA with ivermectin plus albendazole covering ≥85% of the population [21]. In another study, 20 years after cessation of a 5-year DEC-fortified salt programme, five of 32 localities were found to have Mf-prevalence ≥1% [6]. In Leogane, Haiti, sustained transmission has been observed in six villages despite 7 rounds of MDA with an overall prevalence of <1% in an IU [22].

Although the overall Ag-prevalence in children was below 1% in the present study, 17 sites (20% of the total sites) recorded ≥2% Ag-prevalence in children (‘transmission hotspots’). Among these 17 sites, four were from ‘residual hotspots’ and 13 were from the remaining 88 sites (Mf-prevalence <1%, located in the rural setup), suggesting that potential for resurgence exists in these sites. The revised WHO guidelines of 2011 [4] recommend initiation of TAS based on data (prevalence of Mf and/or Ag in population aged >5years) obtained from at least one sentinel and one spot check sites alone. Our findings highlight the risk if such an approach does not recognize the presence of “hotspots” and assumes a homogenous decline of infection in the IU. In this process the chances of overlooking ‘hotspots’ cannot be ruled out. An approach to minimize the risk of overlooking ‘hotspots’ would be, apart from checking prevalence of Mf<1% in the sentinel and spot check sites after fifth MDA [4], transmission (Ag) in children (2–4 years) could also be assessed as recommended in the pre-revised WHO guidelines for TAS [2]. Such an approach could indicate the transmission risk in the sentinel and spot-check sites after fifth MDA before proceeding towards conducting TAS and continuing MDA.

To our knowledge there is no agreed-upon definition for ‘residual/transmission hotspot’. We defined a site as a ‘residual hotspot’ if its Mf-prevalence in the community is ≥1% (irrespective Ag-status in children) or ‘transmission hotspot’ if it has at least one Ag-positive child in 2–8 years, despite the fact that the evaluation unit (in our case the study area) has an overall Mf-prevalence <1%. By following this definition we have identified seven ‘residual’ and 17 ‘transmission’ hotspots in the study area. The population size in these sites varied from 252 to 2318 (mean = 800 & SD = 472). In the site with lowest population, only one Ag-positive child (6 year old) was found among 16 children tested, although the community prevalence of Mf is 0.8% (95% CI: 0.02–4.3%) and Ag-prevalence is 2.4% (95% CI: 0.5–6.8%). In the absence of data on migration status of children (2–8 years), the possibility of acquiring infection elsewhere could not be ruled out. Therefore while declaring a site as ‘transmission hotspot’ based on TAS, it is warranted for the programme managers to ascertain whether the infection is indigenous or acquired elsewhere. Further, it is important that the definition of ‘transmission hotspot’ must also be related to the size/density of population of a site that is large enough to facilitate self-sustaining transmission. In the present study a ‘transmission hotspot’ with a minimum population of 250 could sustain transmission.

Despite reaching <1% Mf-prevalence after seven rounds of MDA with >65% coverage, continued transmission (Ag-prevalence >10% in children aged 3–5 years) was reported from Haiti [22], [23], indicating existence of ‘transmission hotspots’. This corresponds to the definition and existence of ‘transmission hotspots’ in the present study. Adopting an extensive screening exercise as undertaken in this study, to detect “hotspots” would not only be logistically cumbersome but also prohibitively costly. Therefore, further studies are required to identify the determinants as well as methods to rapidly detect such hotspots. A composite index, based on socio-environmental risk factors, was shown to be useful in identifying urban areas with higher infection [24], [25]. Limitation of this approach is obtaining socio-environmental data for all villages/wards in an IU as well as the application of the composite index as such for India, where an organized waste/sewage disposal system does not exist. On the other hand the use of data from remote sensing and geographical information system may be explored as they can easily be obtained. A greater challenge for programmes would be the development of strategies to prevent the emergence of such ‘hotspots’ and site-specific interventions to effectively eliminate them when they persist or appear.

### Distribution of infection by households and sites

Filarial infections are known to be clustered at household level in areas endemic for filariasis [26], [27]. In the present study, despite eight rounds of MDA, we observed clustering of the distribution of infection both at household (Figure 4) and site (Figure 5) levels, i.e. many of the infected persons live in a few households and sites and the remaining are scattered in many households and sites. This finding has important implications for the sampling strategies to be adopted in TAS, particularly when it is based on a cluster design. It is imperative that the sample size in such a design should account for the clustered nature of infections at community level, as has been considered in the revised TAS [4]. This further suggests the need for identifying the factors that lead to heterogeneous distribution of infections. Systematic non-compliance to MDA has been reported to be one of the factors for spatial clustering of infections [22], [23].

### Transmission threshold

Identifying quantitative thresholds in terms of Mf or Ag-prevalence at which MDA programmes can be stopped with minimal risk of resumption of transmission is one of the challenging programmatic issues still facing GPELF [17]. Studies from different parts of the world indicate that the Mf-thresholds varied from 0.4 to 1.0% in thick smears (60 µl blood) [5]–[7] to 0.5% in 1 ml of blood [8]. Ramzy *et al.* [9] have reported that a community Ag-prevalence of <2% could be an alternative threshold to Mf-prevalence of 0.5% at community level. However, the thresholds determined in all the above studies were based on Mf or Ag-prevalence at community level, which is a mixture of old, and new infections. Our extensive analysis of the data from 95 sites throws light on the level of Ag-prevalence in children likely to prevail in the community. Logistic regression analysis of the data from these sites enabled us to predict Ag-prevalence in children, which is 1.6% (95% CI: 0.4–6.2%) for a community Mf-prevalence of 1%. Considering an Ag-prevalence of 1.6% in children aged 2–8 years (equivalent to the recommended Mf-prevalence threshold of ∼1% at community level) as the threshold for stopping MDA, the sensitivity was only 25% whereas the specificity was 94%. However, ROC analysis revealed that both sensitivity (46%) and specificity (81%) could be the maximum at an Ag-prevalence of 0.97%, which corresponds to a community Mf-prevalence of 0.4%. Therefore, an Ag-prevalence of ∼1% in children could be used as threshold for stopping MDA and the corresponding threshold for Mf-prevalence in the pre-TAS exercise could be below 0.4%. The low sensitivity of the model could be due to presence of Ag-positive children (Ag-prevalence range: 1.9–5.6%) in eight of the 95 sites where the observed community Mf-prevalence is ‘zero’. Migration of Ag-positive children from elsewhere could result in such situation, but such possibility is negligible because children (2–8 years) from rural villages are less likely to migrate to other areas. Therefore, presence of false negative Mf-carriers in these eight sites might have contributed for the low sensitivity.

### Limitation

Our conclusions are based on the epidemiological situation obtaining at the time of the conduct of the study and after eight rounds of MDA had been completed. A better assessment of the impact of MDA programme could have been possible if baseline epidemiological situation had been available from the study area. We overcame this limitation to some extent by utilizing the pre-MDA data available from parts of the same IU outside the study area. Another limitation of the study stems from the quality of the MDA programme undertaken in the study area. Although eight MDA rounds were conducted, they were not only spread over 11 years but used two different drug regimens (DEC alone initially and DEC+Albendazole later).

### Implications

The conclusions derived from the study will have implications to the proposed TAS under programme conditions. The results of mass screening support the decision derived from TAS using cluster design based LQAS, making the TAS protocol valid in making the decision on stopping MDA. Application of TAS would be limited in many of the IUs where historical data on compliance are not available, in spite of meeting other two pre-TAS conditions. TAS are essentially sample surveys covering a few sites in an IU. Therefore, the TAS is likely to miss a large number of ‘transmission hotspots’, thereby increasing the risk of resurgence in an IU. The risk of resurgence may be reduced by considering an evaluation unit (EU) smaller (e.g. PHC, health block) than that of an IU. The advantage of having a smaller EU is that it could help in making programme related decision on stopping MDA with the following options: (i) MDA can be stopped in the EU, (ii) in the event of detecting ‘transmission hotspots’, an alternative intervention strategy can be adopted for the entire EU or a targeted approach may be adopted for the ‘transmission hotspots’. However, such an approach would, undoubtedly, greatly increase the operational costs of monitoring and evaluation. Hence, cost-effective and operationally feasible site-specific intervention strategies for tackling the problems related to ‘transmission hotspots’ need to be developed.

### Conclusion

The epidemiological situation after eight rounds of MDA suggests that transmission in the study area as a whole has been reduced to a level <1% (Ag-prevalence) in children, which supports the decision based on TAS for stopping MDA in the study area [4]. However, the study identified several sites with potential for resurgence of infection and provided evidence for the clustering nature of filarial infections. Our analysis suggests that an Ag-level <1% in children (corresponding to a community Mf-prevalence of 0.4%) can be used as a threshold value for stopping MDA. The study highlights the need for identifying factors responsible for the emergence of “transmission hotspots” and adoption of appropriate sampling strategies for the development of evidence-based programmatic decision-making tools. The study also raises important considerations about the size of an EU especially when the IU is as large as a district in India and where it is not possible to survey all the villages to detect ‘hotspots’. Implementation of TAS in the IUs that has already met other two pre-TAS criteria but not having >65% coverage or with no data on coverage would pose challenge for implementing TAS. Finally, we believe that this data set could be utilized in modeling exercises designed to validate, strengthen the monitoring and evaluation efforts of the programme.

## References

[1] World Health Organization (2011) Global Programme to Eliminate Lymphatic Filariasis: progress report on mass drug administration, 2010. Wkly Epidemiol Rec 86: 377–388. 21887884

[2] World Health Organization (2005) Monitoring and epidemiological assessment of the programme to eliminate lymphatic filariasis at implementation unit level. WHO/CDS/CPE/CEE/2005.50.

[3] World Health Organization (2010) Global programme to eliminate lymphatic filariasis – Progress report on mass drug administration in 2009. Wkly Epidemiol Rec 85: 365–372.20853547

[4] World Health Organization (2011) Monitoring and epidemiological assessment of mass drug administration in the global programme to eliminate lymphatic filariasis: a manual for national elimination programmes. WHO/HTM/NTD/PCT/2011.4: 1–79.

[5] Xu B, Cui Z, Zhang Y, Chang J, Zhao Q, et al (1997) Studies on the transmission potential of surviving microfilaraemias after basic control of filariasis. Southeast Asian J Trop Med Public Health 28: 308–313. 9444011

[6] Ramaiah KD, Thiruvengadam B, Vanamail P, Subramanian S, Gunasekaran S, et al (2009) Prolonged persistence of residual Wuchereria bancrofti infection after cessation of diethylcarbamazine-fortified salt programme. Trop Med Int Health 14: 870–876. 19552662 10.1111/j.1365-3156.2009.02307.x

[7] Ramaiah KD, Vanamail P, Yuvaraj J, Das PK (2011) Effect of annual mass administration of diethylcarbamazine and albendazole on bancroftian filariasis in five villages in south India. Trans R Soc Trop Med Hyg 105: 431–437. 21601901 10.1016/j.trstmh.2011.04.006

[8] Michael E, Malecela-Lazaro MN, Kabali C, Snow LC, Kazura JW (2006) Mathematical models and lymphatic filariasis control: endpoints and optimal interventions. Trends Parasitol 22: 226–233. 16564745 10.1016/j.pt.2006.03.005

[9] Ramzy RM, El Setouhy M, Helmy H, Ahmed ES, Abd Elaziz KM, et al (2006) Effect of yearly mass drug administration with diethylcarbamazine and albendazole on bancroftian filariasis in Egypt: a comprehensive assessment. Lancet 367: 992–999. 16564361 10.1016/S0140-6736(06)68426-2

[10] Rajagopalan PK, Das PK, Subramanian S, Vanamail P, Ramaiah KD (1989) Bancroftian filariasis in Pondicherry, south India: 1. Pre-control epidemiological observations. Epidemiol Infect 103: 685–692. 2691269 10.1017/s0950268800031083 PMC2249545

[11] Subramanian S, Pani SP, Das PK, Rajagopalan PK (1989) Bancroftian filariasis in Pondicherry, south India: 2. Epidemiological evaluation of the effect of vector control. Epidemiol Infect 103: 693–702. 2691270 10.1017/s0950268800031095 PMC2249546

[12] Kumar A, Yadav VS, Katoch K, Sachan P (2006) Filariasis in Ghatampur Tahsil of Kanpur Nagar District: indications of high endemicity locus. Journal of Communicable Diseases 38: 155–159. 17370678

[13] Sunish IP, Rajendran R, Satyanarayana K, Munirathinam A, Gajanana A (2001) Immunochromatographic test (ICT) for estimation of true prevalence of bancroftian filariasis in an endemic area in southern India. Trans R Soc Trop Med Hyg 95: 607–609. 11816431 10.1016/s0035-9203(01)90094-x

[14] Steel C, Ottesen EA, Weller PF, Nutman TB (2001) Worm burden and host responsiveness in Wuchereria bancrofti infection: use of antigen detection to refine earlier assessments from the South Pacific. Am J Trop Med Hyg 65: 498–503. 11716104 10.4269/ajtmh.2001.65.498

[15] Watanabe K, Itoh M, Matsuyama H, Hamano S, Kobayashi S, et al (2003) Bancroftian filariasis in Nepal: a survey for circulating antigenemia of Wuchereria bancrofti and urinary IgG4 antibody in two rural areas of Nepal. Acta Trop 88: 11–15. 12943971 10.1016/s0001-706x(03)00157-8

[16] Chandra G, Chatterjee SN, Das S, Sarkar N (2007) Lymphatic filariasis in the coastal areas of Digha, West Bengal, India. Trop Doct 37: 136–139. 17716494 10.1258/004947507781524737

[17] Kyelem D, Biswas G, Bockarie MJ, Bradley MH, El-Setouhy M, et al (2008) Determinants of success in national programs to eliminate lymphatic filariasis: a perspective identifying essential elements and research needs. Am J Trop Med Hyg 79: 480–484. 18840733 PMC2694403

[18] Bal MS, Beuria MK, Mandal NN, Das MK (2009) Antigenemia in young children living in Wuchereria bancrofti-endemic areas of Orissa, India. Trans R Soc Trop Med Hyg 103: 262–265. 18809193 10.1016/j.trstmh.2008.08.006

[19] Lammie PJ, Hightower AW, Eberhard ML (1994) Age-specific prevalence of antigenemia in a Wuchereria bancrofti-exposed population. Am J Trop Med Hyg 51: 348–355. 7943556 10.4269/ajtmh.1994.51.348

[20] Ramaiah KD, Das PK, Vanamail P, Pani SP (2007) Impact of 10 years of diethylcarbamazine and ivermectin mass administration on infection and transmission of lymphatic filariasis. Trans R Soc Trop Med Hyg 101: 555–563. 17374389 10.1016/j.trstmh.2006.12.004

[21] Richards FO Jr, Eigege A, Miri ES, Kal A, Umaru J, et al (2011) Epidemiological and Entomological Evaluations after Six Years or More of Mass Drug Administration for Lymphatic Filariasis Elimination in Nigeria. PLoS Negl Trop Dis 5 10: e1346 doi:10.1371/journal.pntd.0001346. 22022627 10.1371/journal.pntd.0001346 PMC3191131

[22] Boyd A, Won KY, McClintock SK, Donovan CV, Laney SJ, et al (2010) A community-based study of factors associated with continuing transmission of lymphatic filariasis in Leogane, Haiti. PLoS Negl Trop Dis 4: e640. 20351776 10.1371/journal.pntd.0000640 PMC2843627

[23] Washington CH, Radday J, Streit TG, Boyd HA, Beach MJ, et al (2004) Spatial clustering of filarial transmission before and after a Mass Drug Administration in a setting of low infection prevalence. Filaria J 3: 3. 15128461 10.1186/1475-2883-3-3 PMC420477

[24] Bonfim C, Netto MJ, Pedroza D, Portugal JL, Medeiros Z (2009) A socioenvironmental composite index as a tool for identifying urban areas at risk of lymphatic filariasis. Trop Med Int Health 14: 877–884. 19624474 10.1111/j.1365-3156.2009.02317.x

[25] Bonfim C, Alves A, Costa TR, Alencar F, Pedroza D, et al (2011) Spatial analysis and privation index to identify urban areas with a high risk of lymphatic filariasis. Trop Med Int Health 16: 748–755. 21395929 10.1111/j.1365-3156.2011.02758.x

[26] Vanamail P, Subramanian S, Das PK, Pani SP, Bundy DAP (1989) Familial-clustering in Wuchereria bancrofti infection. Tropical Biomedicine 6: 67–71.

[27] Vanamail P, Ramaiah KD, Krishnamoorthy K, Pani SP, Das PK (1992) Distribution of microfilaria carriers and clinical cases of bancroftian filariasis in relation to family size in an urban situation. Tropical Biomedicine 9: 91–98.

